# Female Presidents of the “Royal College of Pathologists”: Their Achievements and Contributions

**DOI:** 10.3389/fmed.2022.861909

**Published:** 2022-04-06

**Authors:** Sarah E. Coupland, Lance N. Sandle, Michael Osborn

**Affiliations:** ^1^Department of Molecular and Clinical Cancer Medicine, University of Liverpool, Liverpool, United Kingdom; ^2^Liverpool Clinical Laboratories, Liverpool University Hospitals NHS Foundation Trust, Liverpool, United Kingdom; ^3^The Royal College of Pathologists, London, United Kingdom; ^4^Department of Cellular Pathology, Charing Cross Hospital, North West London Pathology Hosted at Imperial College NHS Trust, London, United Kingdom

**Keywords:** Royal College of Pathologists, Chemical Pathology, diagnostic histopathology, Dame Barbara Clayton, Dr. Suzy Lishman, Prof. Jo Martin, leadership in Pathology

## Abstract

The Royal College of Pathologists (RCPath) celebrates its Diamond Jubilee in 2022 since its opening by Her Majesty Queen Elizabeth II in 1962. One of the main remits of RCPath is the overseeing of the training of pathologists and scientists working in pathology’s 17 different specialties within the United Kingdom and across the globe. During its 60 years, three female Presidents have been elected: Dame Barbara Clayton (1984–1987), Dr. Suzannah (Suzy) Lishman CBE (2014–2017), and Prof. Joanne (Jo) Martin (2017–2020). Whilst Clayton specialised in Chemical Pathology and its relevance to public health, both Lishman and Martin are diagnostic cellular histopathologists with differing areas of expertise. This article reviews the contributions of these three distinguished and inspirational female pathologists to Pathology (“the science behind the cure”), to healthcare, public health and education, medical research, and to teaching. It highlights their qualities as leaders and mentors for those not only in medicine but in other career settings.

## Introduction

The Royal College of Pathologists (RCPath) is celebrating its 60th anniversary in 2022. The College was founded on June 21st, 1962. It received its Royal Charter in February 1970. Its first substantive premises were at two Carlton House Terrace, a Grade I listed Georgian Townhouse located in the heart of Westminster, London. It was opened on 16th December that year by its Royal patron, Her Majesty Queen Elizabeth II. In November 2018, RCPath moved to its new premises, a bespoke award-winning building designed by Bennetts Associates in Alie Street, Aldgate, East London.

The College is a charity with over 11,000 members worldwide. RCPath oversees the training of pathologists and scientists working in 17 different specialties, including cellular pathology, clinical biochemistry, haematology, medical microbiology and virology, immunology, forensic, and veterinary pathology. Most members are medical, scientific, dental, or veterinary practitioners working mainly in United Kingdom hospitals and universities. The Trustee Board of the College includes its Honorary Officers, comprising amongst others the elected President of the College and three Vice-Presidents (Communications; Teaching and Learning; and Professionalism). The President and Vice-Presidents have 3-year terms, and they undertake these unremunerated roles in addition to their routine diagnostic work. The first President of the College was Sir Roy Cameron, from 1962 to 1966. During its 60 years, three female Presidents have been elected: *Dame Barbara Clayton* (1984–1987), *Dr. Suzannah (Suzy) Lishman CBE* (2014–2017), and *Prof. Joanne (Jo) Martin* (2017–2020). The aim of the following article is to review the contributions of these three distinguished female pathologists to Pathology (“the science behind the cure”), to healthcare, and to medical research and teaching.

## Dame Barbara Evelyn Clayton (1984–1987)

Prof. Dame Barbara Clayton was born in Liverpool, United Kingdom, on September 2nd, 1922, to her parents Constance Evelyn (née Caine) and William Clayton, who then moved to Orpington, London (1). Her father was a food scientist who is renowned for inventing salad cream. Dame Barbara was educated at St Nicholas Preparatory School in Orpington, and subsequently at nearby Bromley County School for Girls, where she was head girl. She then studied medicine at the University of Edinburgh and the Edinburgh Royal Infirmary, qualifying in 1946. Following graduation, she commenced a Ph.D. in the Medical Research Council Clinical Endocrinology Unit in Edinburgh, on the topic of oestrogens (1, 2). After completing this in 1949, Clayton returned to London and became the Holden Research Fellow at St Thomas’s Hospital Medical School for 7 years, before she was promoted to Clinical Lecturer in Chemical Pathology. Her research on hormones – e.g., adrenocorticotropic hormone (ACTH), parathyroid hormone and its interactions with Vitamin D, as well as ascorbic acid metabolism – and the development of new biochemical techniques – brought her well-deserved recognition.

In 1959, she moved to Great Ormond Street Hospital (GOSH) where she was a consultant pathologist and continued to do research, particularly in the genetic metabolic disorders suffered by newborn babies. She pursued a passion for improving childrens’ healthcare and was renowned for her expertise in the diagnosis of phenylketonuria – the special diet that Clayton designed continues to be in common use today (3, 4).

In 1964, Prof. Clayton and others were very concerned with the high levels of lead found in some children’s blood, and this led to her writing a landmark paper entitled “Lead poisoning in children” in 1964 (5), highlighting the detrimental effects lead has on the growth and development of their nervous systems. Whilst a member of the “Royal Commission on Environmental Pollution” in the 1980s, she campaigned for and lobbied the United Kingdom government to enforce a ban on lead in petrol, paint, and other products.

In 1978, after the sudden death of her husband (William [Bill] Klyne), Clayton moved to the new medical school at the University of Southampton as Prof. of Chemical Pathology and Human Metabolism (*its first female Prof.*) and honorary consultant chemical pathologist at the University hospital. She was Dean of Medicine at the University from 1983 to 1986, and honorary consultant chemical pathologist at the Southampton General Hospital. Appointed Emeritus Prof. in 1987, she continued her work on nutrition, but this time looking at the needs of the elderly, particularly those in care homes, about which there was little information and under resourcing.

Over her career, Clayton published about 200 academic papers, which addressed basic and translational research as well as hospital-based and community screening/care of metabolic disorders. She also served on more than 30 expert committees, several of which she chaired. These included the British Nutrition Foundation, the Association of Clinical Biochemists, the Biomedical Sciences Section of the British Association for the Advancement of Science, the Society for the Study of Inborn Errors of Metabolism, the British Nutrition Foundation, and the medical/scientific panel of the Leukaemia Research Fund. Clayton served on Royal Commission on Environmental Pollution from 1981 to 1996 and chaired the enquiry into the Camelford water pollution incident in 1988. The latter dealt with the inadvertent addition of aluminium sulphate to the water supply, raising the concentration to 3,000 times the then admissible level.

**FIGURE 1:**
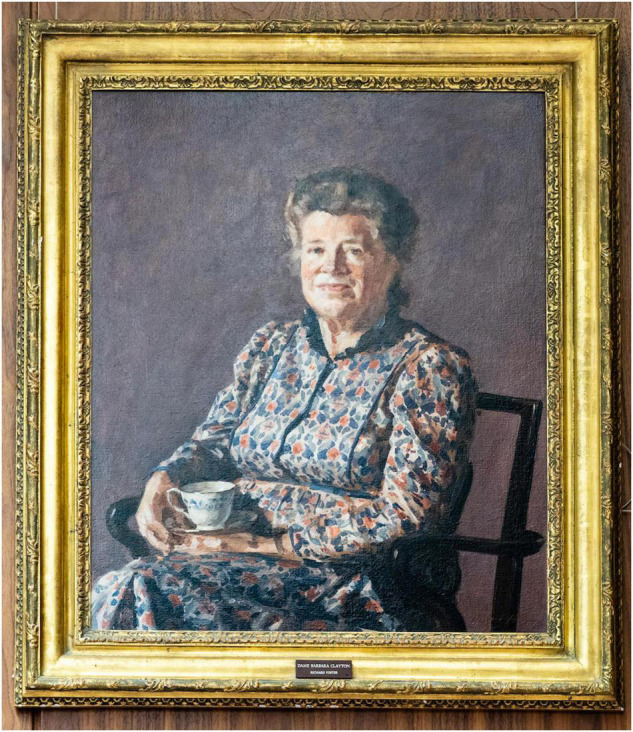
Prof. Dame Barbara Clayton.

Relevant to this article, *Clayton was the first female President of the Royal College of Pathologists* her term being 1984–1987. Her College portrait now located in the new RCPath building in Alie Street shows her not with microscope or a learned tome on her lap but with a simple cup of tea – a symbol of her convivial personality and her ability to solve problems with diplomacy (Figure 1). She was also President of the National Society for Clean Air and Pollution (1995–1997) and the British Dietetic Association (1989–2008).

She received several Honours including a CBE in 1983 and DBE in 1988 for “outstanding contributions on the importance of diet and nutrition and in Chemical Pathology.” In 1999, she was awarded the “British Medical Association’s Gold Medal” for distinguished merit (a rarely awarded honour), and was conferred an Honorary Fellow of the Institute of Biology as well as an Honorary Fellow of the Royal College of Paediatrics and Child Health.

Dame Barbara Clayton was an inspiration and believed that women just had to be better than men to succeed. She was both sociable and private, never shouting about her own achievements.

## Dr. Suzannah (Suzy) Claire Lishman, CBE (2014–2017)

Dr. Suzy Lishman was born in 1967 into a medical family (grandfather and father, both GPs; aunt, respiratory physician; mother and both grandmothers, all nurses), in Beverley in the East Riding of Yorkshire and was educated at Wakefield Girls’ High School, The King’s School Ely and the Neale Wade Community College in March, Cambridgeshire (6). She grew up in Yorkshire and the Fens in England. She was inspired by her aunt Angela, who showed her that women can have good careers in medicine, and so she followed her footsteps to Girton College, Cambridge and the London Hospital Medical College to study Medicine, qualifying in 1992. After completing house jobs in East London, Lishman applied to University College Hospital to specialise in histopathology; she completed this specialist training in 1999. Her first consultant job was at Hinchingbrooke Hospital in Huntingdon, and she moved to Peterborough District Hospital in 2006. The hospitals merged in 2017 to form North West Anglia NHS Foundation Trust. Lishman is currently a consultant cellular pathologist and lead medical examiner at this Trust. She has an interest in colorectal pathology and is pathology lead for the Bowel Cancer Screening Programme.

**FIGURE 2:**
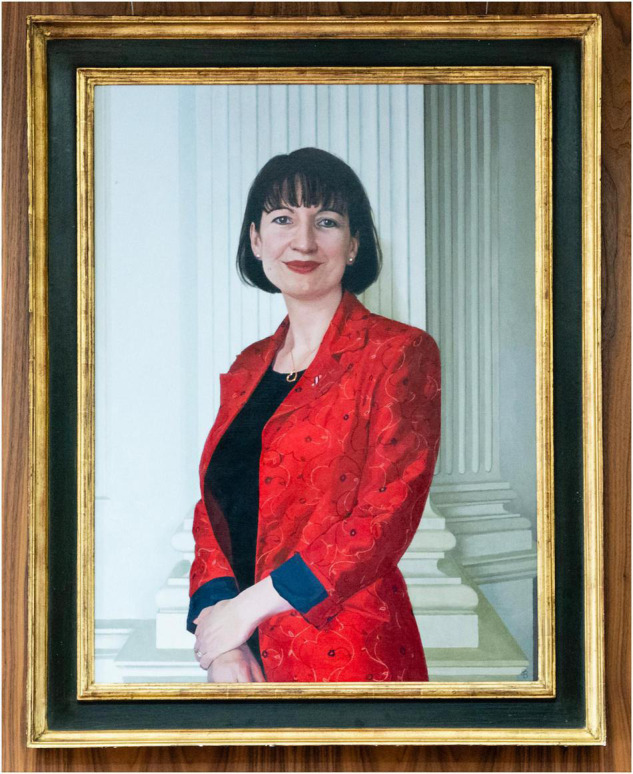
Dr. Suzy Lishman, CBE.

Lishman was involved with the College very early even as a Trainee in Histopathology, attending trainees’ committee meetings. Between 2005 and 2017, she held every honorary office of the Royal College of Pathologists (except Treasurer), as Assistant Registrar (2005–2009), Registrar (2009–2011), Vice-President (Communications; 2011–2014), and President (2014–2017) (Figure 2). During this time, Dr. Lishman raised the profile of the speciality tremendously by introducing public engagement initiatives such as National Pathology Week and International Pathology Day. She has closely collaborated with the Science Museum, Royal Institution, Royal Society, and Cheltenham Science Festival, amongst many other venues and organisations. She has also contributed to numerous television documentaries, talking on a range of topics from the health of Henry VIII to the hidden dangers in the Tudor, Victorian, and Edwardian home. Lishman is active on social media with the Twitter and Instagram handles of “@ilovepathology.” She uses various media to achieve outreach: in particular, Lishman is renowned for the development of “Living Autopsy” events, which involve a talk and simulation about what happens during a post-mortem examination. The format employs a “living model,” who “acts” as a dead body whilst the pathologist talks through how they would perform an autopsy, showing the real instruments used and then drawing on the chest of the model’s body to explain where incisions are made, the location of major organs and what tests would be carried out. The aim of the 60–90 min demonstration is to give a scientifically accurate and sensitive account of this important medical examination. The film of Dr. Lishman performing “The Living Autopsy” has received well over a million views on the RCPath YouTube channel (7). Dr. Lishman has often adapted her Living Autopsy demonstration to incorporate various themes of cultural and local interest. Previous events have explored the bubonic plague, Richard III’s death, and what would happen if one died in space. Her public engagement work led to some amusing situations, including “being filmed for television demonstrating the effect of wearing a tight corset on a male model at Griff Rhys Jones’ London home; performing a virtual brain autopsy at Latitude Festival (complete with blancmange brain); and being interviewed by actor Larry Lamb about the pathology faced by soldiers in WWI trenches). In 2013, she was named one of the fifty most inspirational women in healthcare by the “Health Service Journal,” which described her as the “public face of pathology” and “the most outward facing person from that specialism.” Dr. Lishman was awarded the Royal College of Pathologists’ Medal in 2010 and the Royal Society Kohn Award in 2012 for her public engagement work.

Ultimately, Dr. Lishman was elected President of the Royal College of Pathologists, commencing in November 2014, *as the College’s second female (and youngest) president*. As President, she passionately represented the views of members, working closely with other specialist societies, and forging links with parliamentarians and other policy makers to ensure that pathology is considered in health-related discussions. She continued performing her living autopsies and talking to school groups in between presidential duties. A quote from Lishman is: “One of the joys of being a College officer is working with a diverse group of people from different regions, specialties and professional backgrounds for the benefit of members and patients.”

Following her Presidential term, Dr. Lishman was appointed to (and remains) Chair of the RCPath Medical Examiners’ Committee and national training lead for medical examiners. Medical examiners are senior medical doctors, who are trained in the legal and clinical elements of death certification processes. The Committee overseas a national system of medical examiners that is currently being rolled out in England and Wales to provide much-needed support for bereaved families and to improve patient safety. Dr. Lishman has delivered training to over 1400 medical examiners so far and is currently co-chairing joint training sessions for medical examiners and coroners and organising the second annual medical examiners conference. She is also one of the editors of the first textbook for medical examiners, which will be published in 2022.

In addition to her work on death certification reform, Dr. Lishman is a member of Council of the Royal Veterinary College and chairs their Ethics and Welfare Committee. She has chaired the Scientific Advisory Committee of the charity Bowel Cancer United Kingdom since 2017 and is a trustee of National Enquiry into Patient Outcome and Death (NCEPOD) and the Association for Art History. She has served as a non-executive director of the Medical Protection Society and is on the national Lynch Syndrome Steering group. Dr. Lishman regularly gives talks to schools through “Speakers for Schools” and “Inspiring the Future” and mentors disadvantaged students through the Social Mobility Foundation. She is a regular contributor to several leadership courses, particularly for women.

Dr. Lishman has won several awards: she was nominated one of the top 100 Pathologists of the Power List in 2015 (8, 9) and 2018. In 2018, she also received a CBE for services to pathology. She has an honorary doctorate (DSc) from Swansea University, is an honorary member of the Royal Colleges of Physicians of London, Edinburgh, and Ireland and is an honorary fellow of Girton College, Cambridge. Dr. Lishman was appointed to the David Jenkins memorial Chair in Forensic and Legal Medicine in 2019 and is an honorary fellow of that organisation.

## Prof. Joanne (Jo) Elizabeth Martin (2017–2020)

Prof. Jo Martin was born into a modest background, with a stimulating, happy family in Hertfordshire. Her education was supported by a county scholarship to the local girls’ school. She moved to Uppingham School for A levels for the science teaching, and she applied to Cambridge University for Medicine and was accepted. She was the first of her family to go to university. She qualified *via* Cambridge University and the London Hospital Medical College in 1984 and, following House appointments at Guy’s and then St Thomas’ Hospitals, she returned to the London Hospital to train in pathology. Martin was awarded an MRC Training Fellowship in 1988, an MRC Fellowship in 1990 and Wellcome Trust Advanced Research Training Fellowship in 1991. She gained her MRCPath (as it was then) in 1993. She was awarded a Ph.D at the University of London in 1997 in “The cellular pathology of the lower motor neuron in motor neuron disease,” before becoming established at Queen Mary University of London. She was appointed Clinical Senior Lecturer/Consultant Histopathologist in 1996 and subsequently, Prof. of Neuropathology in 1997 at Queen Mary University of London. She was the only clinician in a major international programme of genetics related to neurodegeneration and neurological disorders, and as part of this designed the “SHIRPA” protocol which has been used in models ever since (10). She has published over 130 papers, including in Nature group and Science journals. She is a practising histopathologist, with a particular expertise in neuromuscular disease of the gut and renal pathology.

Prof. Martin has wide experience of healthcare management and leadership in a range of positions, including Acting Medical Director (January–June 2010), Deputy Medical Director (June 2010–December 2011), Interim Chief Medical Officer (July 2015–January 2016), and Director of Academic Health Sciences at Barts Health NHS Trust. She negotiated the entry of Barts and Queen Mary into UCLPartners, an Academic Health Science partnership, as founding partners. She was Executive lead for both the Clinical Research Network North Thames, and the Collaboration for Leadership in Applied Health Research and Care (CLAHRC) North Thames, involving multiple acute, primary care and third sector organisations and higher education establishments across a very wide complex organisational region. She led education and research across Barts Health NHS Trust, and created App-based training tools for staff, students, patients, and carers, including eCPD which has delivered over 50,000 free modules. She also acquired further degrees at University of London Master’s degree in Leadership (2005), and as part of her involvement in pathology benchmarking, became an Honorary Lecturer in Healthcare Management, Keele University. Additionally, from 2013 to 2016, Prof. Martin was National Clinical Director for Pathology for NHS England.

**FIGURE 3:**
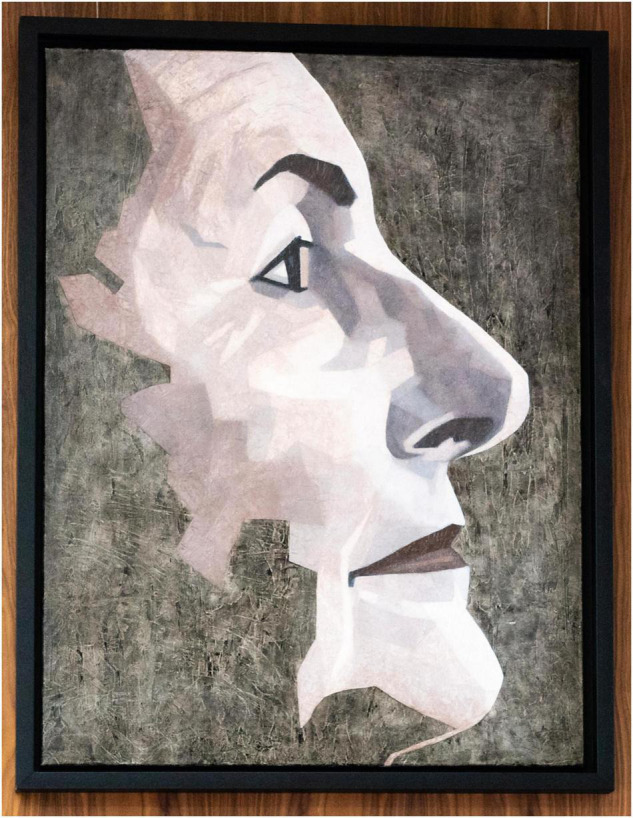
Prof. Jo Martin.

*Prof. Martin was elected the third female President of the Royal College of Pathologists from November 2017 until November 2020* (Figure 3). During her Presidency, she worked across programmes and projects in all the 17 pathology disciplines within the College including genetics, transfusion, digital pathology, data, networks, and with many professional bodies and patient groups. She also visited labs all over the United Kingdom to meet pathologists and learn about their needs and expertise. In particular, she championed expansion of, and support for, workforce, and investment into laboratory information management systems, many of which are obsolete, and roll out of digital pathology. All three of these programmes are now funded and being rolled out, with her support from NHSE/I as National Speciality Advisor, as she Chairs the National Pathology Board and Pathology Workforce Board.

Since March 2021, she has been Director of the Blizard Institute, part of the Whitechapel campus of The Faculty of Medicine and Dentistry of Queen Mary University London and is a world class biomedical research institute that integrates all stages of research from basic science through to clinical studies across a diverse range of fields including genomics, cell biology, translational immunology, neuroscience, and trauma. She has chaired the Research Advisory Board of the Motor Neurone Disease Association since 2018, with involvement in that organisation dating back to 1997. In 2021, she took up post as the Deputy Vice Principal Health at Queen Mary University London.

Finally, Prof. Martin is RCPath lead in a partnership with Health Education England on the innovative Pathology Portal project. The Pathology Portal, to be formally launched in June 2022, will deliver a platform to host high-quality training materials in all pathology disciplines that can be customised to individual needs covering flexible training, return-to-work training, and testing to support learning. The aim is to expand the platform to cover all pathology specialties, providing trainees with an adaptive learning approach, to support development of proficiency in general and specialist areas and to provide flexible and equitable access to content.

For her dedication to Pathology, exceptional service to healthcare, research, and education, Prof. Martin has received numerous awards, including Innovation Trust of the Year NHSIL (2012); Innovation of the Year, Barts Health NHS Trust (2018); she was nominated to be one of the top 100 Pathologists of the Power List in 2018 and was number 3 on this list (11). Her eCPD app won the Education App of the year 2019 at the United Kingdom App awards. She has received the Israel Doniach Lifetime Achievement Award Pathological Society (2018) (12); IBMS Honorary Fellowship (2020; a rare and prestigious honour); Honorary Fellowship of the Royal College of Physicians of Ireland (2020) and Honorary Fellowship of the Faculty of Public Health (2021), in recognition of her work during the COVID pandemic.

## Summary

Each of RCPath’s former female Presidents represent inspirational and formidable women, each having differing strengths, contributing in differing ways to Pathology, healthcare, medical teaching, and research, all with steely determination, stamina, and great leadership skills. Their lifepaths were/are ones of commitment and determination, where they have made the absolute best of the “cards they had been dealt” at each stage and have created clearer paths for those following behind them. They are mentors for future generations of women in not only a career in medicine, but one in all areas of life. In the Box 1 below is a quote from each of them, which may provide guidance for future generations.

**Box 1. Citations from RCPath’s former female Presidents.**

*Prof. Dame Barbara Clayton*: “I usually get my own way when I want it.”

*Dr. Suzy Lishman CBE*: “If you see an inspirational person who’s done what you hope to do, ask if they’ll be your mentor. Don’t be afraid to ask – people are generally pleased to share their experience to help others.”

*Prof. Jo Martin*: “Do whatever you can to make it better – we can all make a positive difference.”

As part of the College’s 60th Birthday year, we celebrate what Clayton, Lishman, and Martin have achieved as Leaders and what they have contributed to this speciality.

## Author Contributions

All authors listed have made a substantial, direct, and intellectual contribution to the work, and approved it for publication.

## Conflict of Interest

The authors declare that the research was conducted in the absence of any commercial or financial relationships that could be construed as a potential conflict of interest.

## Publisher’s Note

All claims expressed in this article are solely those of the authors and do not necessarily represent those of their affiliated organizations, or those of the publisher, the editors and the reviewers. Any product that may be evaluated in this article, or claim that may be made by its manufacturer, is not guaranteed or endorsed by the publisher.

## References

[1] Richmond C. *Lancet Obituary.* (2011) 377:23–9.

[2] Munksroll. *Munks Roll Details for Barbara Evelyn (Dame) Clayton.* London: The Roll of the Royal College of Physicians (2018).

[3] Casemore DP Armstrong M Jackson B Gordon Nichols Thom BT. Screening for cryptosporidium in stools. *Lancet.* (1984) 1:734–5. 10.1016/s0140-6736(84)92245-1 6200740

[4] Clayton BE. Phenylketonuria. *J Med Genet.* (1971) 8:37–40. 10.1136/jmg.8.1.37 5098069 PMC1468963

[5] Moncrieff AA Koumides OP Clayton BE Patrick AD Renwick AG Roberts GE. Lead poisoning in children. *Arch Dis Child.* (1964) 39:1–13. 14160083 10.1136/adc.39.203.1 PMC2019168

[6] Suzy Lishman: grateful to the air bubble. *BMJ.* (2015) 351:h3458. 10.1136/bmj.h3458 26136333

[7] Youtube. *Living Autopsy — Dr Suzy Lishman — Discovery Day at Home.* (2020). Available online at: https://www.youtube.com/watch?v=rGwJQuKZjpI (accessed Sep 26, 2020).

[8] The Pathologist. *The Power List.* (2015). Available online at: https://thepathologist.com/power-list/the-power-list-2015/7-suzy-lishman (accessed January 25, 2022).

[9] Government Digital Service. *”New Year’s Honours 2018” (PDF).* London: Government Digital Service (2017). p. 17.

[10] Rogers DC Fisher EM Brown SD Peters J Hunter AJ Martin JE. Behavioral and functional analysis of mouse phenotype: SHIRPA, a proposed protocol for comprehensive phenotype assessment. *Mamm Genome.* (1997) 8:711–3. 9321461 10.1007/s003359900551

[11] The Pathologist. *The Power List.* (2018). Available online at: https://thepathologist.com/power-list/2018/3-jo-martin (accessed January 25, 2022).

[12] Pathological Society. *Chronological list of Doniach Lecturers.* Edinburgh: Pathological Society (2011).

